# Investigation of Weld Root Defects in High-Power Full-Penetration Laser Welding of High-Strength Steel

**DOI:** 10.3390/ma15031095

**Published:** 2022-01-30

**Authors:** Hengquan Zhang, Meng Jiang, Xi Chen, Lianfeng Wei, Shizhong Wang, Yumo Jiang, Nan Jiang, Zhiyuan Wang, Zhenglong Lei, Yanbin Chen

**Affiliations:** 1 Nuclear Power Institute of China, Chengdu 610213, China; zhanghengquanhit@163.com (H.Z.); wlf21b909141@163.com (L.W.); masc1993@163.com (S.W.); 2 State Key Laboratory of Advanced Welding and Joining, Harbin Institute of Technology, Harbin 150001, China; yumotosjtu@163.com (Y.J.); jiangnanhit@yeah.net (N.J.); wzy4212@163.com (Z.W.); leizhenglong@hit.edu.cn (Z.L.)

**Keywords:** laser welding, high power, single pass, full penetration, welding parameters, root defects

## Abstract

The currently available high-power laser shows promising opportunities for the welding of thick plates in a single pass. However, weld-root defect frequently occurs when a high-power laser is used to join thick plates in a full-penetration mode, which has a significantly adverse effect on the serviceability of the weld joint. The purpose of this work is to understand the defect formation mechanism and reduce these defects through controlling welding parameters. In this study, the characteristics of weld root defects were investigated using a 10 kW fiber laser using a program of experiment and theoretical analysis. The corresponding defect formation mechanisms were discussed based on the bottom molten pool behaviors observed by the high-speed camera. The results showed that there were four types of weld-root appearances as follows with an increase of linear heat input from 300 J/mm to 1000 J/mm: weld-root humping (30 mm/s), sound weld (25 mm/s), weld sagging (20 mm/s) and excessive weld sagging. The remedies for reducing weld-root defects were also presented to obtain sound weld bead by optimizing welding parameters. Weld-root humping was formed due to the quasi-full-penetration keyhole. Weld sagging resulted from the imbalance of the hydrostatic pressure and surface tension in the condition of a through keyhole. It was also found that the sound weld was formed when a through keyhole and a proper molten pool size were obtained. Thus, the state of the keyhole and molten pool geometry were the major factors that affect weld-root defects.

## 1. Introduction

The solid-state laser, fiber laser or disk laser, with the advantages of high brightness, high Rayleigh length and high power output, can produce an ultra-high peak power density of MW/mm^2^ corresponding to a focused electron beam [1,2]. The desirable laser heat source makes laser welding show the characteristics of a large weld aspect ratio, a narrow heat-affected zone, and a high degree of automation over traditional arc welding. Laser welding has become a mature process of fabricating thin components in some manufacturing sectors, such as the automotive sector. Currently, high-power lasers (10–100 kW) are available for welding, which is of considerable interest for the joining of thick components, such as shipbuilding, power plants, pressure vessels, high-speed trains, lifting equipment, wind power towers, and oil/gas pipelines [3,4]. However, defects frequently occur in the welding of thick components using a high-power laser.

Several attempts have been made to use high-power lasers to join thick section components with a single pass [5,6]. Kawahito et al. carried out the research of high-power (10 kW) laser welding in order to clarify the physical phenomena during the process [7]. The results showed that several welding defects such as underfilling, humping, and porosity were generated, and the processing window for production of sound welds was narrow. Thereafter, the formation mechanisms of weld defects were investigated by high-speed camera and X-ray transmission in situ observation. They stated that weld defects could be prevented by selecting the angle of incident laser, defocused positions, and laser spot diameter adequately. Most of the presented research was undertaken on bead-on-plate welding in partial penetration by Kawahito et al. [8,9]. When the high-power state laser was utilized in full-penetration mode welding, some more serious problems arose. Kaplan et al. carried out a full-penetration laser welding process of 16 mm thick stainless steel with a 15 kW fiber laser [10]. They found that, although sound welds could be achieved, heavy spatter ejection along with underfilling and weld sag took place at the top and root sides. Zhang et al. encountered similar problems when a 12 mm thick stainless steel was welded using a 10 kW fiber laser [11]. They stated that root defects frequently occurred in full-penetration mode. A sound full-penetration joint could be achieved only under the conditions of a critical region of welding speed, a proper focal position and a good gas protection. The abovementioned weld-root humping and weld sag, and some other expressions, such as root sag [12], back sag [13], and root droplet [14] comprise just one category of weld-root defects. The aforementioned works also proved that weld-root defects were a crucial issue in single-pass full-penetration welding with a high-power laser.

Attempts were also made to study the effect of welding parameters on weld-root defects and to try to reduce weld-root defects by controlling welding parameters. Shen et al. found that underfilling and back sag were the main restrictions in single-pass laser welding of 10 mm thick 30 CrMnSiA ultra-high-strength steel, especially at the lower welding speeds [13]. Bachmann et al. indicated that a high welding speed was necessary to avoid a weld sag in single-pass hybrid laser welding of 16 mm thick pipe [15]. Frostevarg et al. presented that sufficient laser power could prevent root droplets in single-pass high-power fiber laser welding [14]. Similarly, Pan et al. reported that humping along the bottom surface occurred at low laser power in hybrid laser–MAG welding of 11 mm thick high-tensile-strength steel by a single pass [16,17]. For the root defect formation mechanism, Blecher et al. stated the competition of surface tension and hydrostatic pressure was expected to govern root defect formation [18]. Zhang et al. found that keyhole behaviors also played an important role in determining the root defect formation [12]. According to the above studies, the methods to reduce weld-root defects by controlling welding parameters are inconsistent. Therefore, it is necessary to reveal the defect’s formation mechanism, because it can provide an important guidance on parameter optimization in industry applications of high-power laser welding. Moreover, the mechanism of the root defect formation should also be investigated considering the combined effect of pressure balance and keyhole behaviors.

In this study, single-pass full-penetration welding of 10 mm thick high-strength steel was performed using a 10 kW fiber laser. The correlation of welding parameters and weld-root defects was summarized according to the weld appearance features, and the formation mode of weld-root defects was discussed on basis of bottom molten pool behaviors observed by a high-speed camera. The remedies for reducing weld-root defects were also presented to obtain sound weld bead by optimizing welding parameters.

## 2. Materials and Methods

### 2.1. Materials

The base metal used was 10 mm thick high-strength steel (Q690) plates with dimensions of 100 mm (width) × 200 mm (length). The chemical compositions of the BM are listed in Table 1. Before welding, the surface of the high-strength steel was polished by abrasive paper and wiped with acetone to eliminate the oxides.

### 2.2. Experimental Method

**Figure 1:**
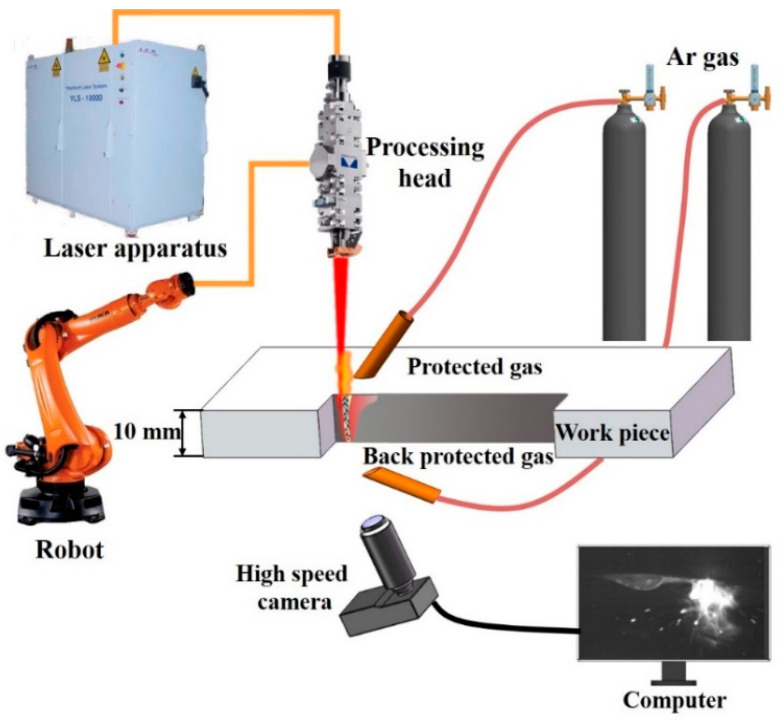
The schematic diagram of experimental set-ups for high power laser welding and observing molten pool behaviors at bottom side.

The schematic diagram of the experimental setup used in this work is illustrated in Figure 1. A continuous wave (CW) fiber laser (YLS-10000 from IPG Photonics) with 1070 nm wavelength and 10 kW maximum output power was used in this experiment. The laser was transmitted through an optical fiber of 200 μm in core diameter to a laser processing head (YW 52 from Precitec Group). The laser processing head was mounted on an industry robot (KR16 from KUKA Robotics). A collimator lens with a focal length of 150 mm and a focusing lens with a focal length of 300 mm were equipped in the laser processing head. The laser beam was focused into a spot with a theoretical diameter of 0.4 mm at the focusing position. After the laser-welding process, the welds were cut perpendicular to the travel direction by electrical discharge machine. Then, metallurgical samples were prepared according to the standard procedure by grinding and polishing. Finally, they were etched with a solution of 5 mL nitric acid and 95 mL ethanol for observation under a light microscope (GX71 from Olympus).

In order to study the formation mechanisms of the weld-root defects in high-power full-penetration laser welding, a CMOS CamRecord 5000 × 2 high-speed camera system (Optronis GmbH, Berlin, Germany) was used in this study. As exhibited in Figure 1, the high-speed camera was set below the work piece in order to observe the bottom molten pool behaviors. The welding phenomena were recorded at a speed of 5000 frames per second. For the purpose of eliminating the interference of strong light, an optical filter with transmittance of 20% was placed in front of the high-speed camera.

## 3. Results and Discussion

### 3.1. Effect of Welding Parameters on Weld-Root Defects

**Figure 2:**
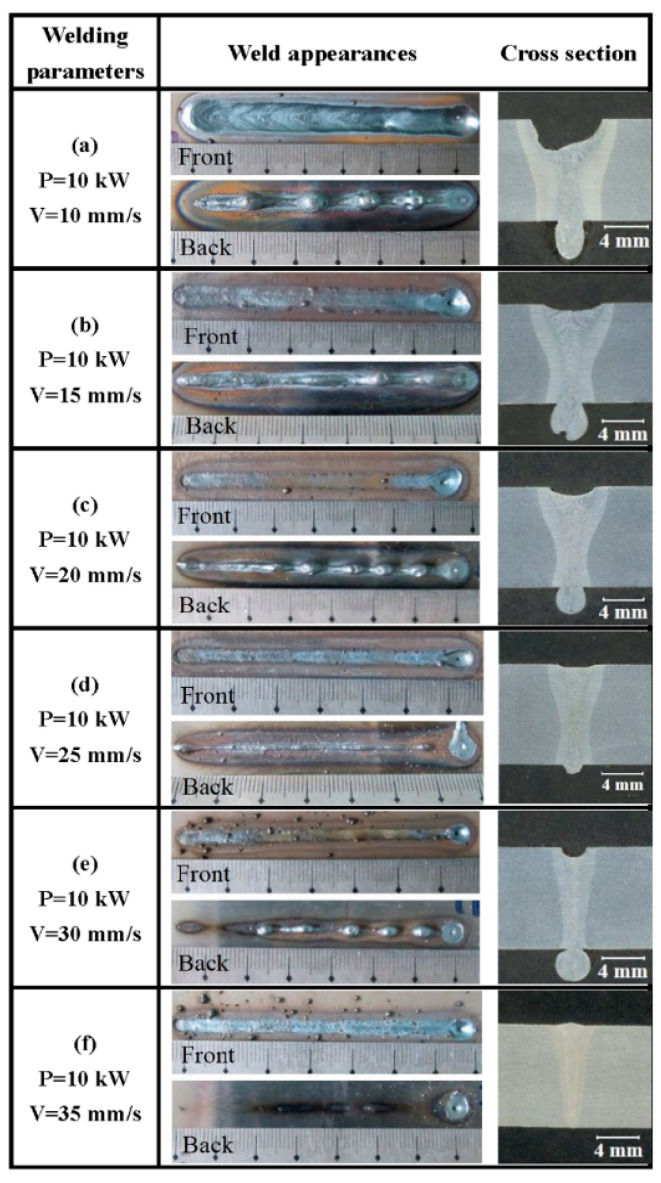
Weld appearances and cross sections of single-pass full-penetration laser-welded high-strength steel at various welding speeds (P—laser power; V—welding speed).

**Figure 3:**
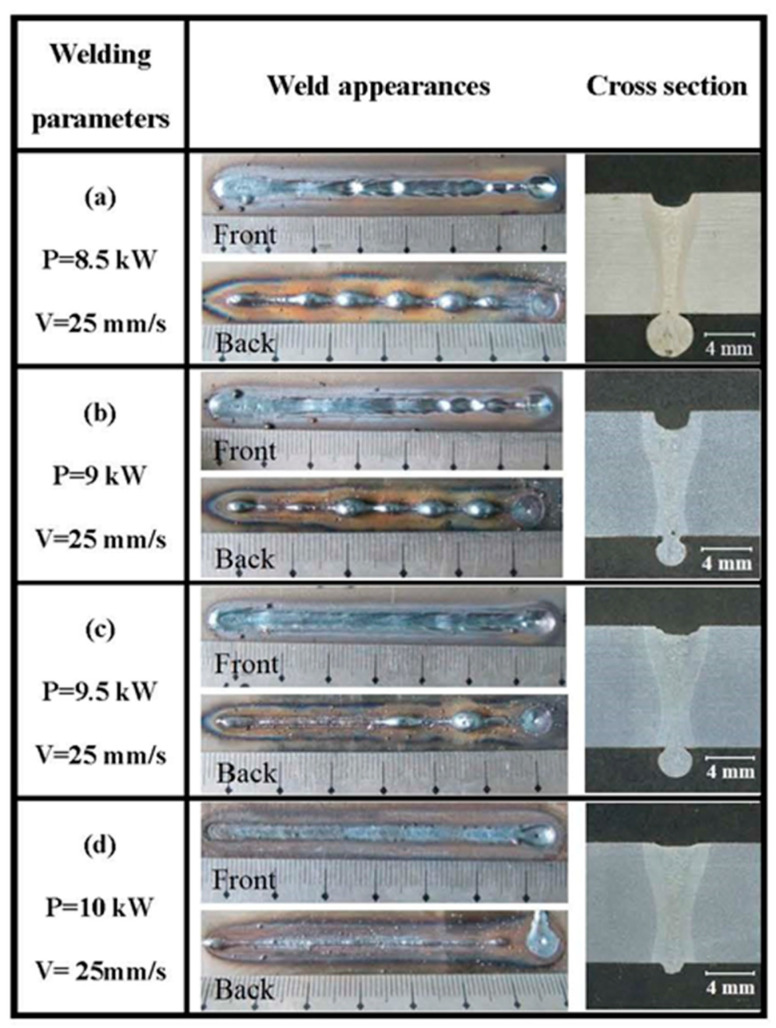
Weld appearances and cross sections of single-pass full-penetration laser-welded high-strength steel with various laser powers (P—laser power; V—welding speed).

In order to clarify the effect of welding parameters on weld-root defects, a series of full-penetration bead-on-plate welding were conducted. The typical weld appearances and cross sections of full-penetration laser-welded high-strength steel for various welding speeds are provided in the Figure 2. The results showed that it was difficult for 10 mm thick high-strength steel to obtain a sound weld appearance using single-pass full-penetration laser welding. Root defects such as humping and sagging usually take place during single-pass full-penetration laser welding. At the same time, underfilling at the top surface was often associated with root sag or humping. When welding speed was 10 mm/s, a wide weld bead was obtained due to the high linear heat input. A deep underfilling was formed and some large melt droplets appeared at the bottom surface. The melt droplets were not independent, and the part between melt droplets was excessive root penetration melt metal. With the welding speed increasing to 15 mm/s or 20 mm/s, both weld width and the underfilling depth decreased. A continuous root sagging appeared, occasionally with some droplets. When the welding speed increased to 25 mm/s, a relatively sound weld was obtained with the smooth weld root appearance. With a further increase in welding speed, a narrow weld bead with deep underfilling and intermittent humps was formed. When the welding speed increased to 35 mm/s, the linear heat input was not enough to achieve full penetration. Based on the present results, the welding speed window of sound weld appearance was very narrow. As shown in Figure 3, the laser power had a similar effect on weld appearances with welding speed. The weld sags also appeared in a wide range of laser power.

**Figure 4:**
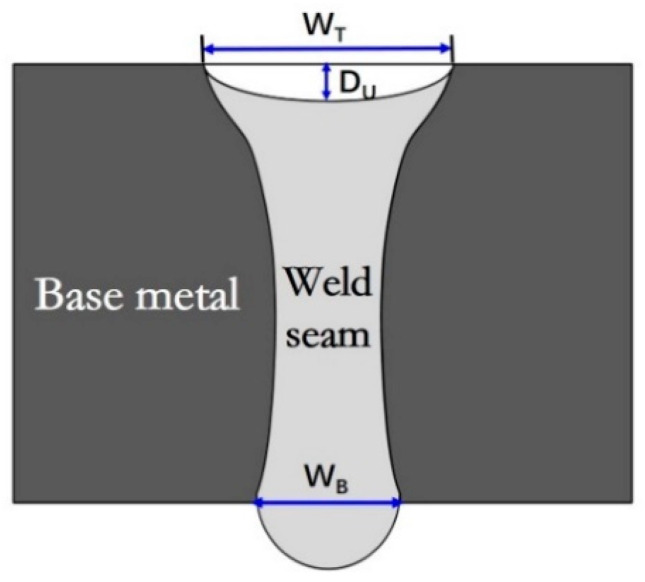
Typical schematic of weld cross-section profile made by single-pass full-penetration welding.

**Figure 5:**
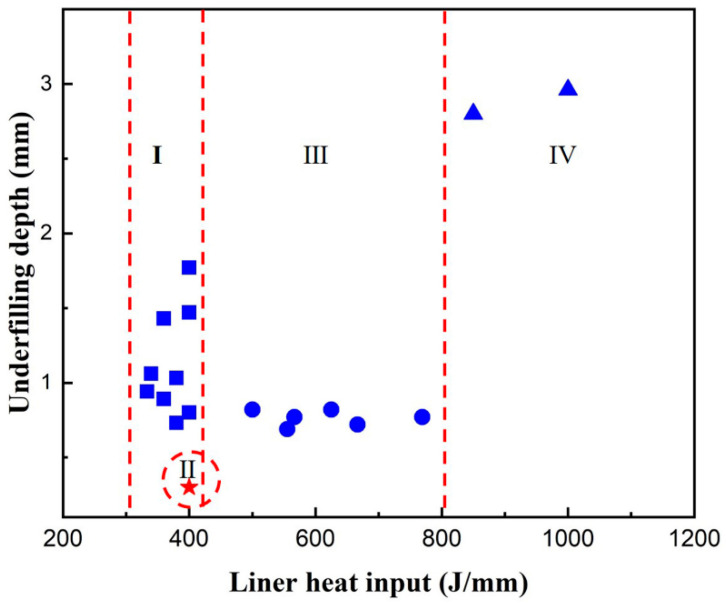
The scatter diagram of Du as a function of linear heat input.

Figure 4 illustrates the typical schematic of weld cross section profile obtained by single-pass laser welding. The weld profile parameters include Wt (the top weld width), Wb (the bottom weld width) and Du (the underfilling depth). The Wt and Wb are affected by welding parameters, and they have a close relationship with the gravity and surface tension, whose balance was the key factor in control of the weld-root appearances. Because underfilling always accompanies with weld sagging, the Du can indicate weld root appearance to some extent. The scatter diagram of underfilling depth as a function of linear heat input was illustrated in Figure 5. The linear heat input could be divided into four regions according to the different underfilling features. The four linear heat input regions correspond to four types of weld-root appearance in full-penetration laser welding. The types of weld-root appearance with the increase of linear heat input are as follows:

- Type I: a narrow weld bead with deep underfilling and intermittent humps at a low linear heat input. This type of weld-root appearance could be named as weld-root humping, which corresponds to Figure 2e;
- Type II: a sound weld bead with the smooth weld-root appearance with a narrow linear heat input window. In addition, there are some limits of welding parameters even within the linear heat input window. This type of weld-root appearance corresponds to Figure 2d;
- Type III: a wider weld bead than Type II; with a small underfilling, a continuous root sagging and occasionally some small droplets. This type of weld-root appearance could be named as weld sagging, which corresponds to Figure 2b or Figure 2c;
- Type IV: a wide weld bead with a deeper underfilling than Type III;, excessive root penetration melt metal and some big melt droplets. This type of weld-root appearance is the excessive Type III, and could be named as excessive weld sagging, which corresponds to Figure 2a.

### 3.2. Effect of Welding Parameters on Bottom Molten Pool Behaviors

**Figure 6:**
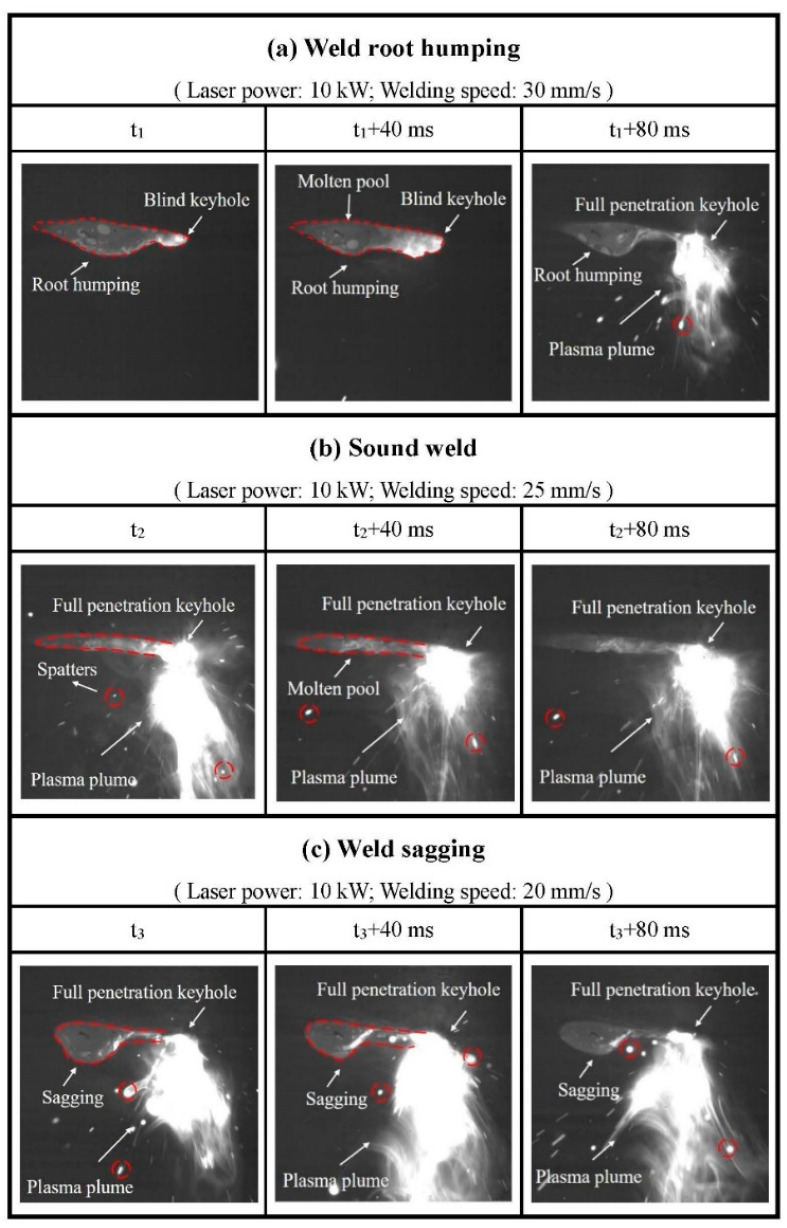
High-speed camera observation results of bottom molten pool behaviors for different types of weld-root defects.

In order to clarify the formation mechanisms of different weld-root defects, the bottom molten pool behaviors at different types of weld-root appearance were observed by the high-speed camera. A constant laser power of 10 kW and three welding speeds, 20 mm/s (weld sagging), 25 mm/s (sound weld), and 30 mm/s (weld-root humping), were used. The corresponding bottom molten pool behaviors are displayed in Figure 6. When laser power was 10 kW and welding speed was 30 mm/s, weld-root humping was obtained. Figure 6a shows the bottom molten pool behaviors when weld-root humping happened. At a certain time, t1, the bottom molten pool could be observed clearly, which means that a full penetration molten pool was achieved. There was a high brightness area in the front of the molten pool, which was the position of the keyhole. However, a blind keyhole was obtained because of its closure at bottom side. A small liquid droplet appeared at the position of the blind keyhole in the bottom molten pool. And a root hump was formed in the middle and rear parts of the molten pool. As time elapsed, the liquid droplet was driven by backward metal flow to the rear part of the molten pool at t1 + 40 ms. At that time, the keyhole was still a blind keyhole. When it came to t1 + 80 ms, the keyhole exit at the bottom side started opening. A plasma plume and some spatters were observed under the bottom molten pool. A root hump was formed in the rear molten pool. In this condition, the keyhole exit at the bottom molten pool surface closed and opened periodically. In the condition of sound weld, as displayed in Figure 6b, the plasma plume could be seen beneath the bottom molten pool all the time during the whole laser welding process. Although the size and intensity of the plasma plume fluctuated constantly and some spatters accompanied, the keyhole exit at the bottom side stayed open. Both molten pool and the keyhole were in full penetration in this condition. A stable, slender, and elongated molten pool was obtained due to the stale open keyhole, resulting in a sound weld appearance. As exhibited in Figure 6c, the plasma plume in the condition of weld sagging displayed a similar behavior with that in the condition of sound weld. The keyhole exit also stayed open throughout the laser welding process. A full-penetration molten pool and keyhole were also achieved. However, an unstable and relatively wider molten pool was obtained. In addition, a big liquid metal sag appeared in the middle and rear part of the molten pool. Hence, high-speed camera observations showed that the keyhole exit at the bottom side was not always kept open in the condition of weld-root humping. However, in the conditions of sound weld and weld sagging, the keyhole exit at the bottom side was kept open during the welding process. Despite the similar keyhole behavior at the bottom side, the molten pool behaviors exhibited big differences in the last two conditions.

### 3.3. Formation Mechanism of Weld-Root Defects

**Figure 7:**
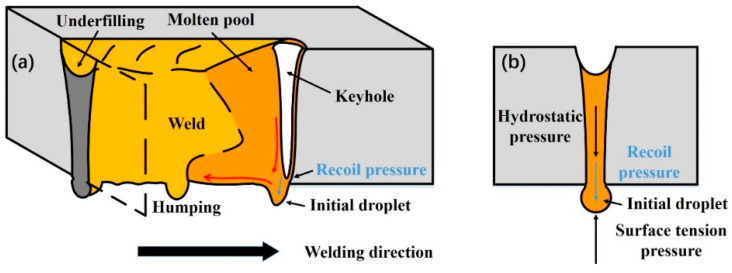
Schematic description of weld-root humping sound weld formation mechanism. (**a**) 3D isometric view; (**b**) Cross-section view.

Weld-root humping was formed at a low linear heat input. It has the features of a narrow weld bead, deep underfilling and intermittent humps. According to the high-speed camera observations, the keyhole exit at the weld-root side was not always kept open in this condition. The full penetration of the liquid weld metal was achieved, while the keyhole was in quasi-full-penetration. As illustrated in Figure 7, full penetration of liquid weld melt with a keyhole is formed. However, the keyhole is a blind keyhole due to the insufficient linear heat input (high welding speed or low laser power). During the keyhole-mode laser welding process, the keyhole is formed because of intense metal evaporation [20,21]. A recoil pressure induced by metal evaporation acts on the liquid melt on the root of the keyhole. Additionally, the liquid melt on the root side of the keyhole also bears the surface tension and the hydrostatic pressure induced by the gravity [22]. The surface tension applies upwards pressure on the liquid melt, while the hydrostatic pressure and recoil pressure apply the downwards pressure on the liquid melt. In general, the surface tension can remain balanced with hydrostatic pressure. However, the liquid metal on the root side cannot remain stable due to the extra recoil pressure. With the driving of hydrostatic pressure and recoil pressure, an initial droplet will build up on the root side of the keyhole. There is a backward flow trend at the bottom molten pool surface during full penetration laser welding, which has been proved by Zhang et al. [23]. The initial droplet was pushed to the molten rear (the solidification front at the same time). Finally, the droplet solidified, resulting in weld-root defects. According to the formation mode, this type of weld-root defect is formed due to the quasi-full-penetration of the keyhole, and has the features of a narrow weld bead with deep underfilling and intermittent humps. Therefore, this type of weld-root defect is called weld-root humping.

**Figure 8:**
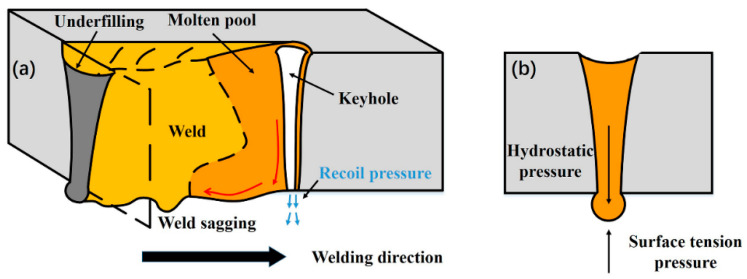
Schematic description of weld sagging sound weld formation mechanism. (**a**) 3D isometric view; (**b**) Cross-section view.

Weld sagging has the features of a wide weld bead, a small underfilling, a continuous root sagging, and some occasional small droplets. It was formed at a high linear heat input. According to the high-speed camera observations, the keyhole exit at the weld-root remained open all the time. It also means that the keyhole was in full penetration in this condition. As shown in Figure 8, a full-penetration molten metal with a through the keyhole is formed due to sufficient linear heat input (low welding speed or high laser power). The metal vapor ejects from the keyhole exit at the root side, and the recoil pressure is released. As for the liquid metal around the keyhole exit at the weld-root side, it is under the surface tension and the hydrostatic pressure caused by gravity. The surface tension is inversely proportional to curvature radius which can be considered as a half of the bottom weld width [24]. When a wide weld is formed due to high linear heat input, the hydrostatic pressure exceeds the surface tension. With the driving of gravity, the molten metal appears as a continuous root sagging. From the high-speed imaging, the bottom molten surface was extremely unstable in this condition. Some droplets also formed occasionally at some locations because of this instability. According to the formation mode, this type of weld-root defect is formed due to the imbalance of the hydrostatic pressure and surface tension in the condition of through keyhole. Because this type of weld-root defect has the features of a wide weld bead with a small underfilling, a continuous root sagging and some occasional small droplets, this type of weld defect is called weld sagging. When the laser input further increases, the weld bead became wider leading to a smaller surface tension. A more excessive root sag appeared. Therefore, the excessive weld sagging has a similar formation mechanism as weld sagging.

**Figure 9:**
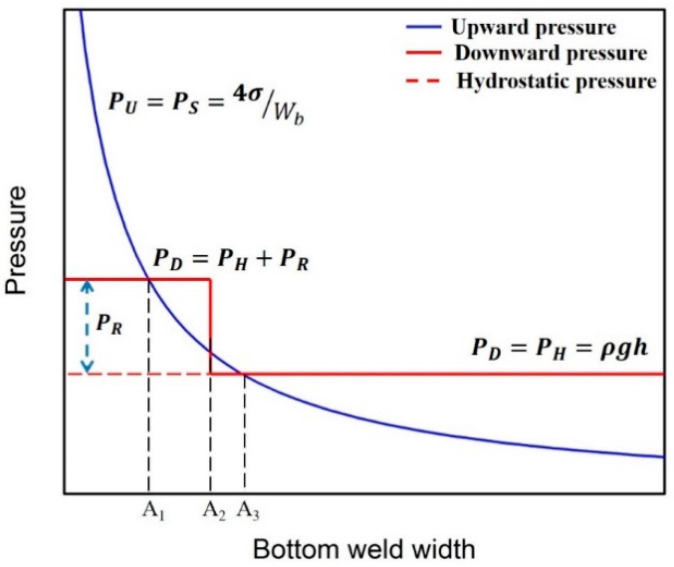
The pressure balance relationship at various weld geometries.

Based on the above discussions, in order to obtain a sound weld, the following two conditions need to be fulfilled at the same time: (1) a full penetration keyhole is formed to release the recoil pressure; (2) the surface tension can balance the liquid metal hydrostatic pressure induced by gravity. Both surface tension and hydrostatic pressure are determined by weld geometries. As shown in Figure 9, the pressure balance is discussed in order to understand the effect of weld geometry on weld-root defects. The upward pressure of liquid metal is mainly provided by surface tension, which is calculated as:

(1) $$P_{U}=P_{S}=\frac{\pi W_{b}\sigma \sin\alpha }{\pi {\left(\frac{W_{b}}{2}\right)}^{2}}=\frac{4\sigma \sin\alpha }{W_{b}}\;$$

where *σ* is the surface tension coefficient, *W_b_* is the bottom weld width and *α* is the contact angle of the solid–liquid interface. The surface tension reaches its maximum value when *α* is 90°. As shown in Figure 9, A2 is the critical value of the bottom weld width for which the keyhole is in full penetration. Therefore, the downward pressure is a piecewise function of the bottom weld width. When the bottom weld width is bigger than A2, the recoil pressure is released, and the downward pressure mainly comes from hydrostatic pressure. The hydrostatic pressure of liquid metal on the weld pool root is

(2) $$P_{D}=P_{H}=\rho gh$$

where *ρ* is the density of the liquid melt, *g* is gravitational acceleration, and *h* is the plate thickness. When the bottom weld width is smaller than A2, the downward pressure is the sum of hydrostatic pressure and recoil pressure.

(3) $$\;P_{D}=P_{H}+P_{R}=pgh+P_{R}$$

**Figure 10:**
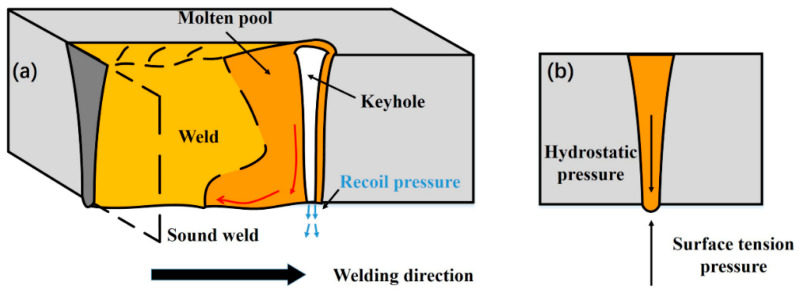
Schematic description of sound weld formation mechanism. (**a**) 3D isometric view; (**b**) Cross-section view.

Based on the relationship between upward pressure and downward pressure shown in Figure 9, the formation mechanisms of weld-root defects are discussed. When the bottom weld width is smaller than A1, the upward pressure provided by surface tension is sufficient to counteract the downward pressure induced by hydrostatic pressure and recoil pressure. In theory, a sound weld bead without weld-root defects can be obtained in this condition. However, it is impossible to achieve such a narrow weld bead in reality. When the bottom weld width is between A1 and A2, the surface tension is sufficient to compensate for the hydrostatic pressure. However, the upward pressure cannot balance the downward pressure because of the extra recoil pressure. In this condition, weld-root humping is formed due to the recoil pressure induced by the blind keyhole. When the bottom weld width is between A2 and A3, a through keyhole is formed and the recoil pressure is released. More importantly, the surface tension is just sufficient for the remaining balance with hydrostatic pressure. A sound weld bead without root defects is obtained in this condition, as displayed in Figure 10. When the bottom weld width is over A3, the hydrostatic pressure exceeds the surface tension. The weld sag is formed in this condition due to the imbalance of surface tension and hydrostatic pressure.

The weld geometries were determined by welding parameters. Therefore, weld-root defects can be prevented by optimization of laser welding parameters in full penetration laser welding. The proper parameters should be chosen to form a through the keyhole and make the bottom weld width between A2 and A3. Therefore, the weld parameters should be adjusted according to the type of root defect. For example, weld-root humping can be prevented by increasing laser power to make a full penetration keyhole. Increasing welding speed may lead to a more serious weld humping. When a weld-root sag is formed, increasing weld speed is the most effective method to obtain a narrower weld bead. Therefore, the state of keyhole and molten pool geometry are the major factors that affect weld-root defects. The strategy of adjusting welding parameters should be determined according to the type of weld-root defect.

## 4. Conclusions

In this study, single-pass full-penetration welding was performed using a high-power laser to investigate the weld-root defect for the welding of thick plates. The types of weld-root defect were summarized according to the weld appearance features. The bottom molten pool behaviors were observed by high-speed camera. The correlation of welding parameters and weld-root defects was summarized, and the corresponding defect formation mechanisms were discussed. The following conclusions can be drawn from this work:

(1) With the increase of heat input from 300 J/mm to 1000 J/mm, four types of weld-root appearances—weld-root humping (30 mm/s), sound weld (25 mm/s), weld sagging (20 mm/s), and excessive weld sagging—were observed sequentially in high-power full-penetration of thick plate;
(2) The keyhole exits at the bottom side were observed to stay open during the whole welding process for conditions of sound weld and weld sagging, while in the condition of weld-root humping, the keyhole exit at the bottom side closed and opened periodically;
(3) Weld-root humping was formed due to the quasi-full-penetration keyhole. Weld sagging resulted from the imbalance of the hydrostatic pressure and surface tension in the condition of a through keyhole. When a weld-root sag is formed, increasing weld speed is the most effective method to obtain a narrower weld bead;
(4) The state of the keyhole and weld geometry were the major factor that affects weld-root defects. The strategy of adjusting welding parameters should be determined according to the type of weld-root defect.

## Figures and Tables

**Table 1:** The chemical composition of high-strength steel (wt%) [19].

Element	Mn	C	Si	S	P	Ni	Nb	Mo	Cr	Ti	Fe
Content	1.28	0.16	0.26	0.003	0.007	0.02	0.027	0.13	0.19	0.018	Bal.

## Data Availability

The data used to support the findings of this study are available from the corresponding author upon request.

## Nomenclature

Abbreviations/symbols/letters	Meaning
CW	Continuous Wave
P	Laser power
V	Welding Speed
Wt	The top weld width
Du	The underfilling depth
*Wb*	The bottom weld width
*σ*	Surface tension coefficient
*α*	Contact angle of the solid-liquid interface
*ρ*	Density of the liquid melt
*g*	Gravitational acceleration
*h*	Plate thickness
